# Antimicrobial Synergistic Effects of Linezolid and Vancomycin with a Small Synthesized 2-Mercaptobenzothiazole Derivative: A Challenge for MRSA Solving

**DOI:** 10.3390/molecules28176348

**Published:** 2023-08-30

**Authors:** Marilena Muraglia, Brigida Immacolata Pia Schiavone, Antonio Rosato, Maria Lisa Clodoveo, Filomena Corbo

**Affiliations:** 1 Dipartimento di Farmacia-Scienze del Farmaco, Università Degli Studi di Bari “A. Moro”, Campus Universitario E. Quagliarello Via Orabona 4, 70125 Bari, Italy; brigidasch@gmail.com (B.I.P.S.); antonio.rosato@uniba.it (A.R.); filomena.corbo@uniba.it (F.C.); 2 Dipartimento Interdisciplinare di Medicina, Università degli Studi di Bari “A. Moro”, Piazza Giulio Cesare 11, 70124 Bari, Italy; marialisa.clodoveo@uniba.it

**Keywords:** *Staphylococcus aureus*, synergism, antibiotic resistance, anti-biofilm, MRSA and MRSE, benzothiazole, small molecule, XTT assay, FICI

## Abstract

Methicillin-resistant *Staphylococcus aureus* (MRSA) emerged as one of the leading causes of persistent human infections and makes it difficult to treat bacteremia, especially with biofilm formation. In this work, we investigated the *in vitro* synergism between Linezolid (LNZ) and Vancomycin (VAN) with a 2-mercaptobenzothiazole derivative, resulting in a new small-molecule antibacterial compound that we named **BTZ2e,** on several clinical MRSA, MRSE (methicillin-resistant *Staphylococcus epidermidis*) and control (ATCC Collection) strains in their planktonic and biofilms cultures. The broth microdilution method evaluated the susceptibility of planktonic cells to each investigated antibiotic combined with **BTZ2e**. The biofilm’s metabolic activity was studied with the XTT reduction assay. As a result, in this study, biofilm formation was significantly suppressed by the **BTZ2e** treatment. In terms of minimal biofilm inhibitory concentration (MBIC), **BTZ2e** revealed an MBIC_50_ value of 32 μg/mL against methicillin-susceptible *S. aureus* (MSSA) and 16 μg/mL against methicillin-resistant *S. aureus* ATCC 43300 biofilms. An inhibition range of 32 μg/mL and 256 μg/mL was registered for the clinical isolates. Interestingly, a synergistic effect (FICI ≤ 0.5) was encountered for the combination of **BTZ2e** with LNZ and VAN on several planktonic and sessile strains. In particular, the best result against planktonic cells emerged as a result of the synergistic association between LNZ and **BTZ2e,** while against sessile cells, the best synergistic association resulted from VAN and **BTZ2e**. The consistent results indicate **BTZ2e** as a promising adjuvant against multi-resistant strains such as MRSA and MRSE.

## 1. Introduction

Antimicrobial multi-drug resistance (AMR) is becoming a serious threat to global public health. The causes of antimicrobial resistance are essentially attributable to either mutations in specific genes or to the acquisition of genes encoding resistant determinants transferred from one microorganism to another. Undoubtedly, the use and misuse of antimicrobial agents can easily promote the selection of resistant pathogens, increasing the prevalence of microorganisms. The World Health Organization (WHO) predicts that by 2050, there could be 10 million deaths worldwide due to infections with multi-drug-resistant bacteria.

This number is expected to increase unless new policies and interventions are implemented. According to the WHO, AMR is among the most relevant global public health problems and causes persistent human infections with severe difficulties or failures in patients’ treatments [1,2,3]. The WHO warns that antibiotic resistance is rising to dangerously high levels in all parts of the world, threatening the ability to cure common infectious diseases that increase morbidity and mortality rates.

### 1.1. MRSA and MRSE Infections: State of the Art

In recent years, the number of antimicrobial agents available and active against resistant bacterial strains has decreased significantly, reducing the available therapeutic options for the treatment of infections caused by these pathogens. Although infections caused by AMR microorganisms are mainly recorded at the hospital level, the number of such infections in the community is increasing [4]. This constitutes a global scientific challenge for the treatment of infections caused by multi-drug-resistance microorganisms such as *Staphylococcus aureus* and emerging new isolates of *Staphylococcus epidermidis*. Methicillin-resistant *S. aureus* (MRSA) and methicillin-resistant *S. epidermidis* (MRSE) are frequent sources of nosocomial infections and higher mortality rates in hospitalized patients, causing serious infections resistant to conventional antibiotic therapies that spreads via medical devices, skin, mucous membranes, and immunocompromised individuals. The major cause of MRSA antibiotic resistance and pervasiveness is to be found in its ability to form biofilm on biotic and abiotic surfaces [5]. Biofilm formation typically plays a crucial role in the development of bacterial infections that are more resistant, leading to significant difficulties in eliminating biofilm-related infections (BAIs) and, in some instances, resulting in chronic and persistent infections. Many factors are involved in biofilm resistance. First, the limitation of the antibiotic entrance is probably due to the presence of a polymerizable mucopolysaccharide, hardly traversable, on the biofilm surface. In addition, its existence in the deepest layers of metabolically inactive cells makes it intrinsically resistant to antibiotics because the accumulation of bacterial cells within the biofilm facilitates the horizontal genetic transfer of the genes responsible for resistance [5]. According to Community Health Assessment, MRSA poses a serious threat due to several factors, such as problems to health and community infrastructure, prevalence, and growing trends in resistance, curability, mortality, preventability, and transmissibility. From an economic perspective, the annual healthcare costs of treating MRSA infections are estimated at USD 3 billion per year [6]. According to the Infectious Diseases Society of America (IDSA), the guidelines for treating MRSA include two commonly used drugs: Vancomycin (VAN) and Linezolid (LNZ) [7]. Antibiotic therapy for the treatment of MRSA infections exposes subjects and patients to the possibility of side effects. VAN, which has been considered the gold standard of therapy for MRSA infections, has been the object of discussion for its slow bactericidal activity and for the emergence of strains resistant to Vancomycin (VRSA) [8]. The tissue penetration of VAN is highly variable and limited in bone tissue, in the lungs, and in cerebrospinal fluid (CSF), and it is nephrotoxic when administered for long periods [9,10]. In *in vitro* studies, it has been observed that VAN is able to induce a greater inhibition of biofilm cells compared to LNZ [11,12]; although LNZ is bacteriostatic, *in vivo*, better efficacy was observed. This result is probably due to its superior pharmacokinetics, its complete bioavailability, and its excellent tissue penetration. Theoretically, an antibiotic could be particularly active against the effects of biofilm when it shows affinity without linking with the matrix and when it can inhibit cells in a slow phase of cell division [11]. Despite the improved *in vivo* efficacy of LNZ, its long-term use is limited by hematologic toxicity from peripheral and optic neuropathy, which are only partially reversible [13,14].

### 1.2. MRSA and MRSE Infections: A Future Perspective

After reviewing the potential side effects of VAN and LNZ as documented in the literature, scientific research programs have suggested an alternative approach to discovering and developing new antibacterial agents for MRSA and MRSE. To minimize negative impacts and lower the necessary dosage, major research programs propose exploring synergistic combinations of both established and newly synthesized antibiotics to combat multi-drug-resistant strains [15,16].

Experimental evidence regarding the combination of LNZ with different antibiotics from various families (glycopeptides, aminoglycosides, rifamycins or quinolones) against *S. aureus* is very encouraging. In contrast, there are few studies regarding therapeutic effects on coagulase-negative staphylococci such as *S. epidermidis* [17,18].

**Figure 1:**
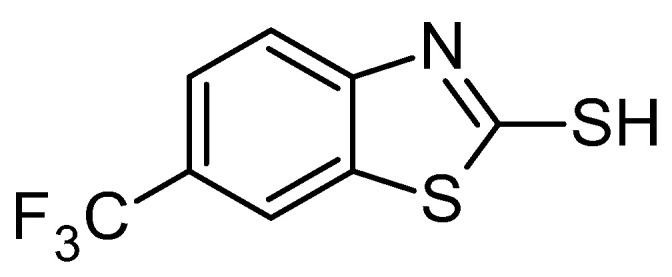
**BTZ2e** (6-fluoromethyl-1,3-benzothiazole-2-thiol).

Hence, in this work, we focused our attention on a small synthesized 2-mercaptobenzothiazole derivative (**BTZ2e**, Figure 1) whose remarkable antimicrobial activity against *S. aureus* and several clinical isolates of *S. aureus* emerged in our previous investigation as a result of our prior research program on antimicrobial agents [19,20,21].

In summary, an interesting positive effect from **BTZ2e** against two vancomycin-resistant clinical isolate strains and MRSA strains, the absence of its cytotoxicity toward MRC-5 cells lines, and the potential involvement of the Nor-A efflux pump in the mechanism of action of compound **BTZ2e** were reported. All this evidence prompted us to assess the efficacious synergistic effects of **BTZ2e** with VAN and LNZ on several clinical MRSA, MRSE and control (ATCC Collection) strains in their planktonic and biofilm cultures with the aim of providing more experimental data for the development of new strategies for treating biofilm MRSA and MRSE infections.

## 2. Results and Discussion

### 2.1. Susceptibility Testing of Planktonic Cells to Antibiotics

The *in vitro* activities of VAN, LNZ and **BTZ2e** against methicillin-sensitive *S. aureus* MSSA, MRSA and MRSE planktonic cells were investigated using the broth microdilution method, and the Minimum Inhibitory Concentration (MIC, μg/mL) values are summarized in Table 1. All MIC determinations were carried out using the NCCLS guidelines [22]. Compound **BTZ2e** was tested in concentrations ranging from 1.56 μg/mL to 100 μg/mL, and LNZ and VAN were tested in concentrations ranging from 0.5 μg/mL to 64 μg/mL. The MIC values of the antibiotics against the quality control strain *S. aureus* ATCC 29,213 were within the range described by CLSI MS-100 [23]. The combined data showed that the investigated compounds exerted inhibitory activity against the tested bacterial strains, with MIC values between 1 and 50 μg/mL. MIC values from 1 to 2 μg/mL for VAN, from 2 to 4 μg/mL for LNZ, and from 3.12 to 12.5 μg/mL for **BTZ2e** were observed. It is noteworthy that compound **BTZ2e** showed promising MIC values against the MRSA strains.

### 2.2. Biofilm Assay

The ability to form biofilms using isolated clinical strains has been proven by Crystal Violet staining, as reported above [24]. The obtained results are shown in Table 2. For this purpose, *S. aureus* ATCC 29,213 was used as a positive control.

The optical density (OD) data reported in Table 2 suggest that all the included strains are moderate to strong biofilm producers. This essential feature allows us to include the studied strains in the biofilm antimicrobial susceptibility testing. Because VAN is a hydrophilic and high molecular-weight molecule, PEG-200 (15% *v*/*v*) was used as a solvent to promote its passage through the matrix of biofilm. A range of 16–256 μg/mL was evaluated.

**Figure 2:**
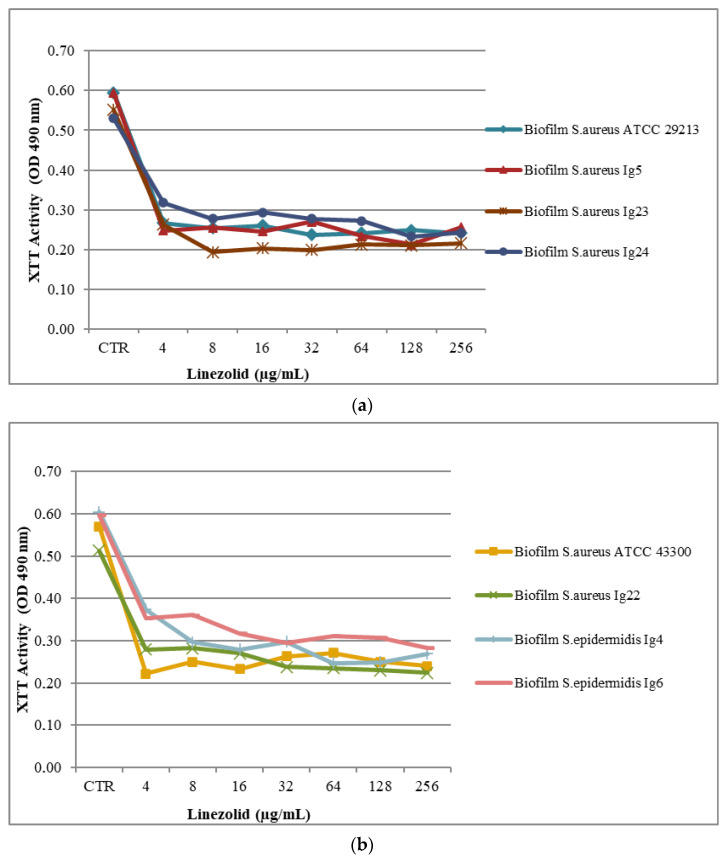
(**a**,**b**). Metabolic activities of biofilms of Staphylococcus spp. exposed to different concentrations of LNZ. Metabolic activity is expressed as optical density value of different concentrations tested on biofilms compared to that for untreated biofilms (control, CTR).

**Figure 3:**
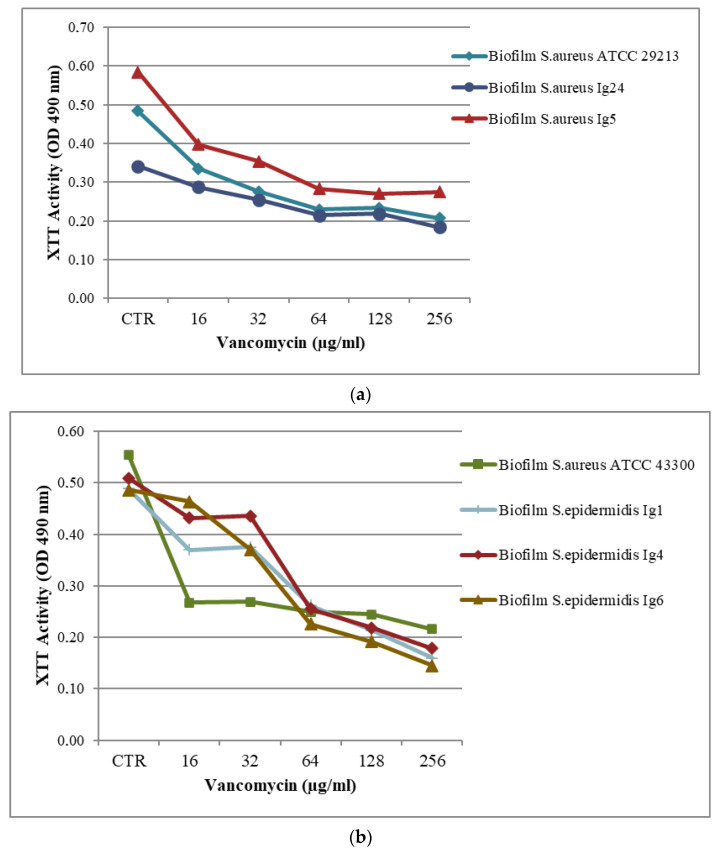
(**a**,**b**). Metabolic activities of biofilms of *Staphylococcus* spp. exposed to different concentrations of VAN. Metabolic activity is expressed as optical density value of different concentrations tested on biofilms compared to that for untreated biofilms (control, CTR).

Antimicrobial activity testing for the biofilm was evaluated in a 96-well plate assay, as reported above [25]. The results are presented in part a and b in Table 3 and Figure 1, Figure 2 and Figure 3a,b. Minimum Biofilm Inhibitory Concentration (MBIC_50_) was defined as the lowest concentration able to inhibit 50% of the biofilm bacterial strain.

The MBIC_50_ value of VAN was 64 μg/mL for *S. aureus* ATCC 29213, *S. aureus Ig5*, *S. epidermidis* and *S. epidermidis Ig4*, *S. epidermidis Ig6*, with percentage reductions of about 50%. VAN MBIC_50_ value was 16 μg/mL against *S. aureus* ATCC 43300. The obtained results showed that for the *S. epidermidis Ig1* biofilm, a percentage reduction of 56% at 128 μg/mL was observed. The *S. aureus Ig22*, *S. aureus Ig23*, and *S. aureus Ig24* biofilms were more resistant, with percentage reductions in the range of 23% and 10% at the highest tested concentration, as reported in part a in Table 3 and Figure 2a,b.

Interesting MBIC_50_ values against *S. aureus* ATCC 43300, *S. aureus* ATCC 29213, *S. aureus Ig5*, and *S. aureus Ig23* biofilms were registered for LNZ at the applied concentration of 4 μg/mL. MBIC_50_ values of 8 μg/mL and 32 μg/mL were observed against the *S. epidermidis Ig4* and *S. epidermidis Ig6* biofilm, respectively, as reported in part b in Table 3 and Figure 3a,b.

**Figure 4:**
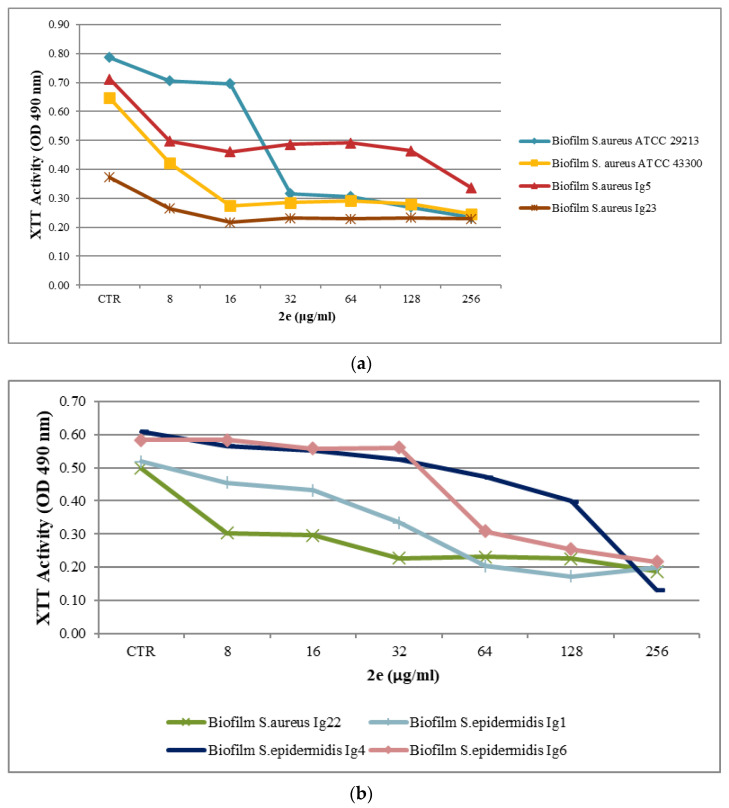
(**a**,**b**). Metabolic activities of biofilms of *Staphylococcus* spp. exposed to different concentrations of **BTZ2e**. Metabolic activity is expressed as the optical density (OD) value of different concentrations tested on biofilms compared to that for untreated biofilms (control, CTR).

The data registered in part c in Table 3 and Figure 4a,b reveal that with regards to MBIC_50_ value, **BTZ2e** was 32μg/mL for *S. aureus* ATCC 29,213 biofilms (MSSA) and 16 μg/mL for S. *aureus* ATCC 43,300 (MRSA). In addition, the combined data showed that **BTZ2e** exerted inhibitory activity (MBIC_50_) against the clinical isolate *S.aureus* ranging from 32 μg/mL to 256 μg/mL.

For the MRSE strains, the *S. epidermidis Ig1*, *S. epidermidis Ig4*, and *S. epidermidis Ig6* biofilm were inhibited between 64 and 256 μg/mL. In the performed test, the final concentration of DMSO in contact with the biofilms was equal to 2.5%, and the DMSO was tolerated up to 10%.

### 2.3. Checkerboard Assay Results

It is worth mentioning that the synergy study involved the combination of two compounds at concentrations below the MIC values. In the present study, the combinations of **BTZ2e** with VAN and LNZ were investigated. For each of the associations, two mixtures were prepared: in the first one, compound **BTZ2e** was diluted in the serial at 40%-20%-10%-5% of the MIC, and the antibiotic was diluted in the serial at 25%-12.5%-6.25%-3.12% of the MIC. The results are shown in Table 4 and Table 5.

Table 4 shows in detail the series of assessments obtained with a concentration of **BTZ2e** between 25% and 3.12% of the MICs, and it shows the two antibiotics in a range of concentrations between 40% and 5% of the MICs.

The reported values highlight an interesting synergistic association with the *S.aureus* strains. Despite this evidence, no synergistic association was found for MRSE. The MICo (MIC of an individual sample), MICc (MIC of an individual sample at the most effective combination) and the FIC (Fractional Inhibitory Concentration) for compound **BTZ2e** ranged from 3.12 to 12.50 μg/mL, from 0.09 to 2 μg/mL, and from 0.03 and 0.25 μg/mL, respectively.

The MICo, MICc, and the FIC for LNZ are between 2 and 4 μg/mL, 0.12 and 1.60 μg/mL, and 0.05 and 0.40 μg/mL, respectively. The MICo, MICc and FIC for the VAN are between 1 and 2 μg/mL, 0.05 and 0.10 μg/mL, and 0.05 and 0.10 μg/mL, respectively.

Table 5 shows the series of assessments obtained with concentrations of **BTZ2e** between 40% and 5% of the MICs, and it shows the two antibiotics in a range of concentrations between 25% and 3.12% of the MICs.

The obtained results show a synergistic action from the studied molecules against the *S.epidermidis Ig4* bacterial strain. No synergistic association was recorded for MRSE.

For the *S.aureus* strains, the MICo, MICc, and FIC of **BTZ2e** are in the range of 3.12 to 12.50 μg/mL, 0.31 to 2.50 μg/mL, and 0.05 to 0.40 μg/mL, respectively. For the *S.epidermidis* strains, the MICo is 50 μg/mL, the MICc is between 10 and 20 μg/mL, and the FIC is between 0.20 and 0.40 μg/mL. The MICo, MICc and FIC for LNZ are between 2 and 4 μg/mL, 0.06 and 1.25 μg/mL, and 0.03 and 0.25 μg/mL, respectively.

The results shown in Table 4 and Table 5 suggest that the best combination of planktonic cells is obtained with a synergy between **BTZ2e** and LNZ, while the combination of **BTZ2e** and VAN seems slightly effective. In addition, by evaluating the FICI values, it can be observed that the mixture is more effective when compound **BTZ2e** is used in the serial from 25% to 3.12%.

Concerning synergistic effects on the biofilm, the following associations were investigated: **BTZ2e** with VAN and **BTZ2e** with LNZ. For each association, two mixtures were prepared: compound **BTZ2e** was diluted in the serial at 40%-20%-10%-5% of the MIC, and the antibiotics were diluted in the serial at 25%-12.5%-6.25%-3.12% of the MICs. The results obtained are shown in Table 6 and Table 7.

Table 6 shows in detail the MBICs values for the synergies between compound **BTZ2e** (concentration range of 25–3.12%) and the antibiotics (concentration range of 40–5%). The MBICo, MBICc, and the FIC values for **BTZ2e** are between 32 and 128 μg/mL, 2 and 128 μg/mL, and 0.03 and 0.25 μg/mL, respectively. Regarding the combination effect of **BTZ2e** with LNZ, the MBICo, MBICc, and FIC values ranged from 4 to 32 μg/mL, from 0.80 to 6.40 μg/mL, and from 0.20 to 0.40 μg/mL, respectively.

The association of **BTZ2e** with VAN produced MBICo, MBICc, and FIC in ranges of 16 to 256 μg/mL, 0.60 and 51.20 μg/mL and 0.05 and 0.40 μg/mL, respectively. The *S. aureus Ig23* strain was excluded from the study investigating the synergy between VAN and **BTZ2e** because it was not possible at the highest concentrations tested to detect 50% inhibition for both compounds. *S. epidermidis Ig1* and *S. aureus Ig24* were excluded from the association study with LNZ as MBIC_50_ was not detected.

Table 7 shows the second set of experiments conducted with concentrations of **BTZ2e** between 40% and 5% of the MBICs, and it shows the two antibiotics in concentrations between 25% and 3.12% of the MBICs. For the *S.aureus* strains, the MBIC_O_, MBICc and the FIC for **BTZ2e** are between 16 and 512 μg/mL, 0.80 and 51.20 μg/mL and 0.05 and 0.10 μg/mL, respectively. The MBICo, the MBICc and the FIC for LNZ are between 4 and 32 μg/mL, 1 and 2 μg/mL, and 0.03 and 0.25 μg/mL. The MBICo, MBICc and FIC for VAN ranged from 16 to 256 μg/mL, from 0.50 to 64 μg/mL and from 0.03 to 0.25 μg/mL, respectively.

The experimental data reported in Table 6 and Table 7 suggest that the potentially useful combinations of **BTZ2e** with VAN are still the best option with **BTZ2e** serial dilutions from 40% to 5%. The decreased percentage of **BTZ2e** produced a lower synergistic efficacy toward the biofilm bacteria. Nevertheless, the association with LNZ in both mixtures tested is scarce.

## 3. Materials and Methods

### 3.1. Bacterial Strains and Culture Conditions

The bacterial strains used in this study are reported in Table 1. Two bacterial strains from American Type Culture Collection (ATCC, Rockville, MD, USA) and seven derived from clinical isolation were included: *S. aureus* ATCC 29,213 (MSSA), *S. aureus* ATCC 43,300 (MRSA), *S. aureus Ig5* (MRSA), *S. aureus Ig22* (MRSA), *S. aureus Ig23* (MRSA), *S. aureus Ig24* (MRSA), *S. epidermidis Ig1* (MRSE), *S. epidermidis Ig4* (MRSE), and *S. epidermidis Ig6* (MRSE). All the isolates were from patients admitted to the Department of Medical Sciences University “A. Moro”, Bari, Italy. The isolation and identification procedures were conducted at the Hygiene Section of the Department. Mueller–Hinton broth (MHB; Oxoid) was adjusted to contain 20 mg/mL Ca^2+^ and 10 mg/mL Mg^2+^. Stock solutions were maintained at −80 °C in nutrient broth (Muller–Hinton II Broth, MHBII, Becton Dickinson, Pont-de-Claix, France) containing 15–20% glycerol (Oxoid, Rodano, Italy) until used. All strains were stored at −20 °C in glycerol stocks and were subcultured on Muller–Hinton agar plates (Oxoid, Rodano, Italy) to ensure viability and purity before the beginning of the study. The bacterial species were cultured on Mueller–Hinton agar (MHA, Oxoid), and each bacterial suspension was composed of 2–3 colonies of each strain, taken from an MHA plate and dissolved in 2 mL of MHB (Mueller–Hinton Broth, Sigma-Aldrich, St. Louis, MO, USA). Cultures were grown on MHBII from 100 μL of frozen culture and incubated aerobically for approximately 4 h at 37 °C.

### 3.2. Test Compounds

Antimicrobial agent Vancomycin (VAN) was purchased from Sigma Aldrich (St. Louis, MO, USA), and Linezolid (LNZ) was supplied by Pfizer (Rome, Italy). **BTZ2e** was freshly synthesized and characterized at the Department of Pharmacy of University “A. Moro” of Bari. **BTZ2e** purity was analytically estimated to be > 99% by performing elemental analysis as reported in Franchini et al. [21].

### 3.3. Planktonic Susceptibility Testing

The Minimal Inhibitory Concentrations (MICs, measured in µg/mL) of the tested compounds were determined *in vitro* using the broth microdilution method, which follows the guidelines set by the Clinical and Laboratory Standards Institute (CLSI, M7-A6, 2003). This method has been reported in previous studies, with some minor adjustments [19,20,21,22,25]. The absorbance of the cellular suspensions, calibrated at a wavelength of 625 nm using the spectrophotometric method (Thermo Spectronic, Genesys 20, Segrate (MI), Italy), should be 0.08 to 0.12 for the 0.5 McFarland standard, corresponding to approximately 1 × 10^8^ CFU (Colony Forming Unit)/mL. To prepare the inoculum, we diluted it to a ratio of 1:100 by adding 100 µL of inoculum to 9.9 mL of MHB. This resulted in a final inoculum value of about 1 × 10^6^ CFU/mL. We then pipetted 20 µL of this dilution into each well. Prior to use, we prepared stock solutions of the solubilized and diluted drugs according to protocol M100-S17 and then plated them in two-fold serial dilutions in the test medium [26]. In each well, 200 µL of these solutions were added. In order to ensure that the solvent did not have any negative impact on the growth of bacteria, a control test was conducted where the medium was combined with ethanol at its highest concentration. Several wells containing only inoculated broth as control growth were prepared. After incubation for 24 h at 37 ± 1 °C, the last well containing no microbial growth was recorded to show the MIC in µg/mL. The MIC was determined by using an antibacterial assay repeated twice in triplicates.

### 3.4. Biomass Assay

The amount of biomass present in the biofilms was measured using the Crystal Violet staining method, which was adapted from [24]. Briefly, the 24 h biofilms formed within the 96 wells were fixed with 250 µL of 98% CH_3_OH per well for 15 min. Next, the plates were emptied and allowed to air dry for 20 min. Following that, the bacteria that had been fixed were stained for 5 min with 200 µL of Crystal Violet (CV) per well. Excess stain was rinsed off by pipetting. After the plates were air-dried, the dye bound to the adherent cells was resuspended with 200 µL of 33% (*v*/*v*) glacial acetic acid per well. The optical density (OD) of the obtained solution was measured at 570 nm using a microtiter plate reader, and biofilm mass is presented as OD570 values. To avoid erroneous outcomes, control experiments were conducted to investigate any possible interaction between the tested media and the polyester construction of the plates with CV [24].

### 3.5. Culture of Biofilms and Susceptibility Testing to Antimicrobial Compounds

Biofilms were developed according to the procedure of Stepanović et al. with a few modifications [24,25]. The bacterial strains were streaked from frozen cultures and grown overnight at 37 °C on a Tryptic soy agar plate (TSB, Merck KGaA, Darmstadt, Germany) with 2% (*w*/*v*) glucose. A number of colonies were dissolved in TBS with 2% (*w*/*v*) glucose until reaching a density of approximately 10^5^ CFU/mL. Briefly, 200 μL of each bacterial suspension was transferred in a 96-well microtiter plate (IWAKI, Japan) to induce biofilm formation. Next, the plates were placed on a horizontal shaker and incubated aerobically for 4 h at 37 °C. Once the incubation was complete, the suspension was delicately removed and replaced with sterile medium. The plates were incubated for 24 h at 37 °C to obtain mature biofilms [25]. Each bacterial strain was tested twice in triplicate.

Following incubation, the plate was rinsed twice with phosphate-buffered saline (PBS) to remove slackly attached planktonic cells. Subsequently, a serial dilution of antimicrobial compound was added to each well. TSB with 2% (*w*/*v*) glucose was used to perform all dilutions. The solution of VAN was obtained in distilled water and tested in concentrations ranging from 256 to 16 μg/mL. LNZ was solubilized in DMSO 100% and tested in concentrations ranging from 256 to 2 μg/mL. Solutions of the tested compound **BTZ2e** were obtained in DMSO 100% at concentrations ranging from 256 to 8 μg/mL. The biofilms thus formed were incubated for 18 h at 37 °C on a horizontal shaker, and control antibiotic-free biofilms were included in each experiment.

The XTT assay was used to quantify the number of viable cells in each of the wells following antimicrobial compound treatment in comparison with biofilms performed in the presence of TSB (control). This method has been used extensively for the quantification of bacterial biofilm, as reported in our previous work [25]. It measures the reduction of a tetrazolium salt (2,3-bis[2-methoxyloxy-4-nitro-5-sulfophenyl]-2H-tetrazolium-5-carboxanilide (XTT), Sigma-Aldrich, USA) by metabolically active cells to colored water-soluble formazan derivative that can be easily quantified colorimetrically [24]. To prepare the XTT solution (1 mg/mL), it was briefly mixed with PBS and filtered through a 0.22 µm pore size filter for sterilization. In each assay, a menadione solution (FluKa, Sigma-Aldrich, USA) (0.4 mM), previously prepared in DMSO, was added. Following antimicrobial compound exposure, the plate was rinsed with PBS to remove loosely attached cells, dried in an inverted position at 37 °C for 20 min, and then 180 µL of PBS and 20 µL of the XTT-menadione solution (12.5 times the volume of XTT solution was mixed with 1 volume of menadione solution) were added to each well, and the plate was incubated for 2 h at 37 °C on a horizontal shaker in the dark [26]. Once the specified time had passed, the wells were subjected to centrifugation, and the resulting supernatant (100 μL) was moved to a fresh microwell plate. The reduction in XTT (oxidative activity) was then measured at 490 nm using aPerkin-Elmer Wallac Victor3 microplate reader. The MBIC_50_, (Minimal Biofilm Inhibitory Concentration) was defined as the lowest concentration able to inhibit the biofilm cells to 50%. The MBIC_50_ was determined by using an antibacterial assay repeated twice in triplicates.

### 3.6. Microdilution Checkerboard Technique

To assess the *in vitro* synergistic effect of VAN, LNZ, and BTZ2e on the MSSA, MRSA, and MRSE bacterial strains, the combination assays utilized the checkerboard method as reported by Rosato et al. [27,28]. Susceptibility was evaluated in a 4-well by 4-well chequerboard format in 96-well microtiter plates [29,30]. Four planktonic cell antimicrobial agents were prepared in fourfold serial dilutions across rows and columns, each well contained 50 µL of drug dilutions and 100 µL of standardized inoculum (10^6^ CFU/mL), and inhibitory endpoints were assessed by XTT reduction assay (25 μL of XTT-menadione solution, XTT (µmg/mL), Men (µmM)) [31]. In the first step of our tests, the serial dilutions ranged from 40% to 5% of MIC values for antibiotics (LNZ and VAN) and from 25% to 3.12% of MIC values of **BTZ2e**. In the second step, higher percentages of **BTZ2e** (40–5%) were used, and lower percentages of the two antibiotics (25–3.12%) were used.

To test the effectiveness of antimicrobial agents against biofilm, we prepared twofold serial dilutions of the drugs. Each well contained 100 µL of the diluted drug. We also used the same serial dilutions for planktonic cells. The inhibitory endpoints were evaluated by performing an XTT reduction assay on the biofilm. In our experimental protocol, the substance combinations were analyzed by calculating the FIC index (FICI) as follows: FIC of the investigated antibiotic agents plus FIC of **BTZ2e**. The analysis of the combinations of the substances was carried out through the calculation of the FIC index (FICI: Fractional Inibitory Concentration):

FICI = (MIC A + B/MIC A) + (MIC B + A/MIC B)

where A and B represent the two antibiotics. The FIC index (FICI) is the synergy value expressed by each combination; the lower its value, the lower the amounts of the two substances needed to induce synergy. Overall, the FICI values had a synergistic effect when ≤0.5; an additive effect when >0.5; and an antagonistic effect when >1 [32].

The combination of the two components can be shown graphically in a Cartesian diagram by applying the isobole method [33].

## 4. Conclusions

Antimicrobial resistance (AMR) has become a serious threat to public health globally. In recent years, the number of available antimicrobial agents against resistant microorganisms has decreased, reducing the therapeutic options available for the treatment of infections caused by these pathogens. Although infections with AMR microorganisms are recorded mainly at the clinical level, infections in the community are increasing, with significant consequences for public health costs and global health. This constitutes a global scientific challenge for the treatment of infections caused by multi-drug-resistant microorganisms such as *S. aureus* and emerging new isolates of *S. epidermidis.* Vancomycin (VAN) and Linezolid (LNZ) have been suggested as standard antibiotics for the treatment of MRSA and MRSE bacteremia by the Infectious Diseases Society of America (IDSA). Anyway, the use of VAN and LNZ remain plagued by serious adverse effects. We agree with this line of research since we were already aware of the remarkable antimicrobial activity of **BTZ2e**, a small synthesized 2-mercaptobenzothiazole derivative (Figure 1), against *S. aureus* and several clinical isolates of *S. aureus.* In this work, we decided to explore the potential synergy between **BTZ2e** and the antibiotics VAN and LNZ. The goal of the present study was to comprehensively identify a **BTZ2e** concentration that can act synergistically with VAN or LNZ for the treatment of infections caused by MRSA and MRSE. To this purpose, several clinical MRSA, MRSE (Methicillin-resistant Staphylococcus epidermidis) and control (ATCC Collection) strains in their planktonic and biofilm cultures were studied by using the broth microdilution method to evaluate the susceptibility of planktonic cells and checkerboard assays to confirm the synergy of each investigated antibiotic with **BTZ2e**. The findings of the present study highlight the potential effects of the synergistic combination of **BTZ2e** with the antibiotics VAN and LNZ on the growth of MRSA and MRSE. The discussed experimental data show that the best combination of planktonic cells is obtained with the synergy of **BTZ2e** and LNZ, while the combination of **BTZ2e** with VAN seems to be ineffective.

In conclusion, all these discoveries suggest that **BTZ2e** has promise as a highly potent anti-MRSA and anti-MRSE agent in combination with VAN and LNZ for the control and prevention of MRSA infection and colonization. Therefore, the present study could be a starting point to better investigate the mechanism of the inhibitory effect of the studied combinations on biofilm formation and their effects on cell membrane permeability.

## Figures and Tables

**Table 1:** Bacterial strains used in this study. Susceptibility of selected *S. aureus* and *S. epidermidis* strains to antibiotics Vancomycin (VAN), Linezolid (LNZ) and compound **BTZ2e**.

MIC (μg/mL)			
Bacterial Strain	VAN	LNZ	BTZ2e
*S. aureus* ATCC 29213	1	4	3.12
*S. aureus* ATCC 43300	1	2.5	12.5
*S. aureus Ig5*	2	4	6.25
*S. aureus Ig22*	1	4	3.12
*S. aureus Ig23*	2	2	3.12
*S. aureus Ig24*	1	2	3.12
*S. epidermidis Ig1*	2	2	50
*S. epidermidis Ig4*	2	2	50
*S. epidermidis Ig6*	2	2	50

MIC (Minimum Inhibitory Concentration) values determined by broth microdilution assay.

**Table 2:** Quantification of biomass formation by Crystal Violet staining.

Bacterial Strain	OD (570 nm)	Description	Categories
*S. aureus* ATCC 29213	2.30	strong biofilm producer	3
*S. aureus* ATCC 43300	1.43	moderate biofilm producer	2
*S. aureus Ig5*	1.82	moderate biofilm producer	2
*S. aureus Ig22*	2.40	strong biofilm producer	3
*S. aureus Ig23*	1.65	moderate biofilm producer	2
*S. aureus Ig24*	1.64	moderate biofilm producer	2
*S. epidermidis Ig1*	2.35	strong biofilm producer	3
*S. epidermidis Ig4*	2.26	strong biofilm producer	3
*S. epidermidis Ig6*	1.44	moderate biofilm producer	2
Negative control	0.49		

**Table 3:** (**a**). Antibacterial susceptibilities of *Staphylococcus* spp. determined using the XTT method, and VAN biofilm cell reduction percentages at the evaluated concentrations. (**b**). Antibacterial susceptibilities of *Staphylococcus* spp. determined using the XTT method, and LNZ biofilm cell reduction percentages at the evaluated concentrations. (**c**). Antibacterial susceptibilities of Staphylococcus spp. determined using the XTT method, and **BTZ2e** biofilm cell reduction percentages at the evaluated concentrations.

a		
Bacterial Strain	VAN	
	MBIC_50_ (μg/mL)	%
*S. aureus* ATCC 29213	64	52
*S. aureus* ATCC 43300	16	51
*S. aureus Ig5*	64	51
*S. aureus Ig22*	R	23
*S. aureus Ig23*	R	10
*S. aureus Ig24*	256	46
*S. epidermidis Ig1*	128	56
*S. epidermidis Ig4*	64	50
*S. epidermidis Ig6*	64	53
**b**		
**Bacterial Strain**	**LNZ**	
	**MBIC_50_ (μg/mL)**	**%**
*S. aureus ATCC* 29213	4	55
*S. aureus ATCC* 43300	4	64
*S. aureus Ig5*	4	58
*S. aureus Ig22*	4	53
*S. aureus Ig23*	4	52
*S. aureus Ig24*	64	48
*S. epidermidis Ig1*	R	30
*S. epidermidis Ig4*	8	51
*S. epidermidis Ig6*	32	50
**c**		
**Bacterial Strain**	**BTZ2e**	
	**MBIC_50_ (μg/mL)**	**%**
*S. aureus* ATCC 29213	32	60
*S. aureus* ATCC 43300	16	58
*S. aureus Ig5*	256	53
*S. aureus Ig22*	32	54
*S. aureus Ig23*	16	43
*S. aureus Ig24*	R	38
*S. epidermidis Ig1*	64	60
*S. epidermidis Ig4*	256	78
*S. epidermidis Ig6*	128	56

MBIC: Minimum Biofilm Inhibitory Concentration; R: Resistant. Results are representative of at least three separate experiments. The MBIC_50_ for biofilms is based on the lowest drug concentration that produces a 50% inhibition of the metabolic activity of the untreated growth control.

**Table 4:** Association of **BTZ2e** (dilution range 25–3.12%) and antibiotics (dilution range 40–5%).

	LNZ				VAN			
Sample	MICo	MICc	FIC	FICI	MICo	MICc	FIC	FICI
***S. aureus* ATCC 29213**								
**BTZ2e**	3.12	0.39	0.12	0.22	3.12	0.39	0.12	0.17
**Antibiotic**	4	1.60	0.10		1	0.05	0.05	
***S. aureus* ATCC 43300**								
**BTZ2e**	12.50	1.56	0.12	0.17	NS			
**Antibiotic**	2.20	0.12	0.05					
***S. aureus Ig5***								
**BTZ2e**	6.25	0.39	0.06	0.26	6.25	0.78	0.12	0.22
**Antibiotic**	4	0.80	0.20		1	0.10	0.10	
***S. aureus Ig22***								
**BTZ2e**	3.12	0.09	0.03	0.13	NS			
**Antibiotic**	4	0.40	0.10					
***S. aureus Ig23***								
**BTZ2e**	3.12	0.09	0.03	0.43	NS			
**Antibiotic**	2	0.80	0.40					
***S. aureus Ig24***								
**BTZ2e**	6.25	0.19	0.03	0.23	6.25	1.56	0.25	0.30
**Antibiotic**	2	0.40	0.20		2	0.10	0.05	

MICo: MIC of an individual sample; MICc: MIC of an individual sample at the most effective combination; FIC: Fractional Inhibitory Concentration; FICI: FIC of antibiotic + FIC of **BTZ2e**. Fractional Inhibitory Concentration (FIC) and FIC Index (FICI); NS: No synergistic combination. Concentrations are expressed as μg/mL.

**Table 5:** Association of **BTZ2e** (dilution range 40–5%) and antibiotics (dilution range 25–3.12%).

	LNZ				VAN			
Sample (μg/mL)	MICo	MICc	FIC	FICI	MICo	MICc	FIC	FICI
***S. aureus* ATCC 29213**								
**BTZ2e**	3.12	0.31	0.10	0.22	NS			
**Antibiotic**	4	0.50	0.12					
***S. aureus* ATCC 43300**								
**BTZ2e**	12.50	1.25	0.20	0.22	NS			
**Antibiotic**	2.50	1.25	0.12					
***S. aureus Ig5***								
**BTZ2e**	6.25	0.31	0.05	0.30	NS			
**Antibiotic**	4	1	0.25					
***S. aureus Ig22***								
**BTZ2e**	3.12	0.31	0.10	0.13	NS			
**Antibiotic**	4	0.12	0.03					
***S. aureus Ig23***								
**BTZ2e**	3.12	1.25	0.40	0.65	NS			
**Antibiotic**	2	0.50	0.25					
***S. aureus Ig24***								
***BTZ2e***	6.25	0.31	0.05	0.08	6.25	2.50	0.40	0.43
***Antibiotic***	2	0.06	0.03		1	0.03	0.03	
***S. epidermidis Ig4***								
***BTZ2e***	50	10	0.20	0.22	50	20	0.40	0.43
***Antibiotic***	2	0.25	0.12		2	0.06	0.03	

MICo: MIC of an individual sample; MICc: MIC of an individual sample at the most effective combination; FIC: Fractional Inhibitory Concentration (see text); FICI: FIC of antibiotic + FIC of **BTZ2e**. Fractional Inhibitory Concentration (FIC) and FIC Index (FICI); NS: No synergistic combination. Concentrations are expressed as μg/mL.

**Table 6:** Synergistic effects of **BTZ2e** (dilution range of 25–3.12%) and antibiotics (dilution range of 40–5%) on biofilm.

	LNZ				VAN			
Sample(μg/mL)	MBICo	MBICc	FIC	FICI	MBICo	MBICc	FIC	FICI
***S. aureus* ATCC 29213**								
**BTZ2e**	32	4	0.25	0.65	NS			
**Antibiotic**	4	0.80	0.40					
***S. aureus* ATCC 43300**								
**BTZ2e**	NS				16	0.50	0.03	0.08
**Antibiotic**					16	0.60	0.05	
***S. aureus Ig5***								
**BTZ2e**	256	8	0.03	0.43	NS			
**Antibiotic**	4	1.60	0.40					
***S. aureus Ig22***								
**BTZ2e**	32	8	0.25	0.45	32	8	0.25	0.45
**Antibiotic**	32	6.40	0.20		256	51.20	0.20	
***S. aureus Ig24***								
**BTZ2e**	NE				512	128	0.25	0.35
**Antibiotic**					256	25.60	0.10	
***S. epidermidis Ig1***								
**BTZ2e**	NE				64	2	0.03	0.43
**Antibiotic**					128	51.20	0.40	
***S. epidermidis Ig4***								
**BTZ2e**	NS				256	64	0.25	0.65
**Antibiotic**					64	25.60	0.40	
***S. epidermidis Ig6***								
**BTZ2e**	NS				128	8	0.06	0.26
**Antibiotic**					64	12.80	0.20	

MIBCo: Minimum Biofilm Inhibitory Concentration (MIC) of an individual sample; MICc: Minimum Biofilm Inhibitory Concentration (MIC) of an individual sample at the most effective combination; FIC: Fractional Inhibitory Concentration (see text); FICI: FIC of antibiotic + FIC of BTZ2e. Fractional Inhibitory Concentration (FIC) and FIC Index (FICI); NS: No synergistic combination. Concentrations are expressed as μg/mL. NE: Not evaluated.

**Table 7:** **BTZ2e** (dilution range 40–5%) and antibiotics (dilution range 25–3.12%).

	LNZ				VAN			
Sample(μg/mL)	MBICo	MBICc	FIC	FICI	MBICo	MBICc	FIC	FICI
***S. aureus ATCC 29213***								
**BTZ2e**	NS				32	1.60	0.05	0.30
**Antibiotic**					64	16	0.25	
***S. aureus ATCC 43300***								
**BTZ2e**	NS				16	0.80	0.05	0.08
**Antibiotic**					16	0.50	0.03	
***S. aureus Ig5***								
**BTZ2e**	256	12,80	0.05	0.30	256	25.60	0.10	0.35
**Antibiotic**	4	1	0.25		64	16	0.25	
***S. aureus Ig22***								
**BTZ2e**	NS				32	3.20	0.10	0.35
**Antibiotic**					256	64	0.25	
***S. aureus Ig24***								
**BTZ2e**	ND				512	51.20	0.10	0.16
**Antibiotic**					256	16	0.06	
***S. epidermidis Ig1***								
**BTZ2e**	ND				64	3.20	0.05	0.17
**Antibiotic**					128	16	0.12	
***S. epidermidis Ig4***								
**BTZ2e**	256	12.80	0.05	0.30	256	25.60	0.10	0.13
**Antibiotic**	8	2	0.25		64	2	0.03	
***S. epidermidis Ig6***								
**BTZ2e**	128	51.20	0.40	0.43	128	6.40	0.05	0.30
**Antibiotic**	32	1	0.03		64	16	0.25	

MIBCo: Minimum Biofilm Inhibitory Concentration (MIC) of an individual sample; MICc: Mini-mum Biofilm Inhibitory Concentration (MIC) of an individual sample at the most effective com-bination; FIC: Fractional Inhibitory Concentration (see text); FICI: FIC of antibiotic + FIC of BTZ2e. Fractional Inhibitory Concentration (FIC) and FIC Index (FICI); NS: No synergistic combination. Concentrations are expressed as μg/mL. NE: Not evaluated.

## Data Availability

Not applicable.

## Sample Availability

Not available.

## References

[1] https://www.who.int/news/item/09-12-2022-report-signals-increasing-resistance-to-antibiotics-in-bacterial-infections-in-humans-and-need-for-better-data.

[2] (EARS-Net) 2017. https://www.ecdc.europa.eu/en/antimicrobial-resistance/surveillance-and-disease-data/report.

[3] Howden B.P., Davies J.K., Johnson P.D.R., Stinear T.P., Grayson M.L. (2010). Reduced vancomycin susceptibility in *Staphylococcus aureus*, including vancomycin-intermediate and heterogeneous vancomycin-intermediate strains: Resistance mechanisms, labora-tory detection, and clinical implications. Clin. Microbiol. Rev..

[4] O’Neill J. (2016). Tackling Drug-Resistant Infections Globally: Final Report and Recommendations the Review on Antimicrobial Resistance.

[5] Cascioferro S., Carbone D., Parrino B., Pecoraro C., Giovannetti E., Cirrincione G., Diana P. (2021). Therapeutic strategies to counteract antibiotic resistance in MRSA biofilm-associated infections. ChemMedChem.

[6] King M.D., Humphrey B.J., Wang Y.F., Kourbatova E.V., Ray S.M., Blumberg H.M. (2006). Emergence of community-acquired methicillin-resistant Staphylococcus aureus USA 300 clone as the predominant cause of skin and soft-tissue infections. Ann. Intern. Med..

[7] Liu C., Bayer A., Cosgrove S.E., Daum R.S., Fridkin S.K., Gorwitz R.J., Kaplan S.L., Karchmer A.W., Levine D.P., Murray B.E. (2011). Clinical practice guidelines by the infectious diseases society of America for the treatment of methicillin-resistant Staphylococcus aureus infections in adults and children: Executive summary. Clin. Infect. Dis..

[8] Nandhini P., Kumar P., Mickymaray S., Alothaim A.S., Somasundaram J., Rajan M. (2022). Recent Developments in Methicillin-Resistant Staphylococcus aureus (MRSA) Treatment: A. Review. Antibiotics.

[9] Altowayan W.M., Mobark M.A., Alharbi A., Alduhami A.A., Rabbani S.I. The influence of vancomycin on renal functions, the predictors and associated factors for nephrotoxicity. PLoS ONE.

[10] Jian Y., Lv H., Liu J., Huang Q., Liu Y., Liu Q., Li M. (2020). Dynamic Changes of Staphylococcus aureus Susceptibility to Vancomycin, Teicoplanin, and Linezolid in a Central Teaching Hospital in Shanghai, China, 2008-2018. Front Microbiol..

[11] Wilcox M.H., Kit P., Mills K., Sudgen S. (2001). In situ measurement of linezolid and vancomycin concentrations in intravascular catheter-associated biofilm. J. Antimicrob. Chemother..

[12] El-Azizi M., Rao S., Kanchanapoom T., Khardori N. (2005). *In vitro* activity of vancomycin, quinupristin/dalfopristin, and linezolid against intact and disrupted biofilms of staphylococci. Ann. Clin. Microbiol. Antimicrob..

[13] Oehadian A., Santoso P., Menzies D., Ruslami R. (2022). Concise Clinical Review of Hematologic Toxicity of Linezolid in Multidrug-Resistant and Extensively Drug-Resistant Tuberculosis: Role of Mitochondria. Tuberc. Respir Dis..

[14] Kawasuji H., Nagaoka K., Tsuji Y., Kimoto K., Takegoshi Y., Kaneda M., Murai Y., Karaushi H., Mitsutake K., Yamamoto Y. (2023). Effectiveness and Safety of Linezolid Versus Vancomycin, Teicoplanin, or Daptomycin against Methicillin-Resistant *Staphylococcus aureus* Bacteremia: A Systematic Review and Meta-Analysis. Antibiotics.

[15] Valderrama M.-J., Alfaro M., Rodríguez-Avial I., Baos E., Rodríguez-Avial C., Culebras E. (2020). Synergy of Linezolid with Several Antimicrobial Agents against Linezolid-Methicillin-Resistant Staphylococcal Strains. Antibiotics.

[16] Liu C., Bayer A., Cosgrove S.E., Daum R.S., Fridkin S.C., Gorwitz R.J., Kaplan S.L., Karchmer A.V., Levine D.P., Murray B.A. (2011). Clinical practice guidelines by the Infectious Diseases Society of America for the treatment of methicillin-resistant Staphylococcus aureus infections in adults and children. Clin. Infect. Dis..

[17] Chen H., Li L., Liu Y., Wu M., Xu S., Zhang G., Qi C., Du Y., Wang M., Li J. (2018). *In vitro* activity and post-antibiotic effects of linezolid in combination with fosfomycin against clinical isolates of *Staphylococcus aureus*. Infect. Drug Resist..

[18] Lee Y.C., Chen P.Y., Wang J.T., Chan S.D. (2019). A study on combination of daptomycin with selected antimicrobial agents: *In vitro* synergistic effect of MIC value of 1mg/L against MRSA strains. BMC Pharmacol. Toxicol..

[19] Armenise D., Carocci A., Catalano A., Muraglia M., Defrenza I., De Laurentis N., Rosato A., Corbo F., Franchini C. (2013). Synthesis and Antimicrobial Evaluation of a New Series of N-1, 3-Benzothiazol-2-ylbenzamides. J. Chem..

[20] Defrenza I., Catalano A., Carocci A., Carrieri A., Muraglia M., Rosato A., Corbo F., Franchini C. (2015). 1, 3-Benzothiazoles as Antimicrobial Agents. J. Heterocycl. Chem..

[21] Franchini C., Muraglia M., Corbo F., Florio M.A., Di Mola A., Rosato A., Matucci R., Nesi M., Van Bambeke F., Vitali C. (2009). Synthesis and Biological Evaluation of 2-Mercapto-1,3-benzothiazole Derivatives with Potential Antimicrobial Activity. Arch. Pharm..

[22] NCCLS (2003). Methods for Dilution Antimicrobial Susceptibility Tests for Bacteria that Grow Aerobically.

[23] (2021). Performance Standards for Antimicrobial Susceptibility Testing, 31st ed.

[24] Stepanović S., Vuković D., Hola V., Di Bonaventura G., Djukić S., Ćirkovic I., Ruzicka F. (2007). Quantification of biofilm in microtiter plates: Overview of testing conditions and practical recommendations for assessment of biofilm production by staphylococci. APMIS.

[25] Schiavone B.I., Rosato A., Muraglia M., Gibbons S., Bombardelli E., Verotta L., Franchini C., Corbo F. (2013). Biological evaluation of hyperforin and its hydrogenated analogue on bacterial growth and biofilm production. J. Nat. Prod..

[26] (2007). Performance Standards for Antimicrobial Susceptibility Testing.

[27] Kuhn D.M., Balkis M., Chandra J., Mukherjee P.K., Ghannoum M.A. (2003). Uses and limitation of the XTT assay in studies of Candida growth and metabolism. J. Clin. Microbiol..

[28] Rosato A., Sblano S., Salvagno L., Carocci A., Clodoveo M.L., Corbo F., Fracchiolla G. (2020). Anti-Biofilm Inhibitory Synergistic Effects of Combinations of Essential Oils and Antibiotics. Antibiotics.

[29] Tian F., Baoping L.B.J., Jinhua Y., Guizhi Z., Yang C., Yangchao L. (2009). Antioxidant and antimicrobial activities of consecutive extracts from Galla chinesis: The polarity affects the bioactivities. Food Chem..

[30] Hübner N.-O., Matthes R., Koban I., Rändler C., Müller G., Bender C., Kindel E., Kocher T., Kramer A. (2010). Efficacy of Chlorhexidine, Polihexanide and Tissue-Tolerable Plasma against Pseudomonas aeruginosa Biofilms Grown on Polystyrene and Silicone Materials. Skin Pharmacol. Physiol..

[31] Cocchietto M., Skert N., Nimis P.L., Sava G. (2002). A review on usnic acid, an interesting natural compound. Naturwissenschaften.

[32] Kim S., Greenleaf R., Miller M.C., Satish L., Kathju S., Ehrlich G., Post J.C., Sotereanos N.G., Stoodley P. (2011). Mechanical effects, antimicrobial efficacy and cytotoxicity of usnic acid as a biofilm prophylaxis in PMMA. J. Mater. Sci. Mater. Med..

[33] Tunney M.M., Ramage G., Field T.R., Moriarty T.F., Storey D.G. (2004). Rapid colorimetric assay for antimicrobial susceptibility testing of Pseudomonas aeruginosa. Antimicrob. Agent Chemother..

